# Multicomponent Analysis in Static and Flow Systems Using Digital Filters

**DOI:** 10.6028/jres.093.034

**Published:** 1988-06-01

**Authors:** Steven D. Brown, Todd Q. Barker, Harlan R. Wilk, Stephen L. Monfre

**Affiliations:** Department of Chemistry and Biochemistry, University of Delaware, Newark, DE 19716

## 1. Multicomponent Analysis as a Routine Analytical Tool

Many samples require rapid quantitative analysis of several components without taking the time required for separation of the components either from each other, or from the sample matrix. This need is especially critical for samples whose existence in some detection zone is transient, either because of some chemical reaction or because of sample transport rates through the analysis manifold. Mathematical analysis of data collected on such systems offers a practical route to the enhancement of detector selectivity without significant degradation of detection limits.

## 2. A Brief Review of Digital Filters

A set of techniques which is particularly suited to use in the analysis of transient responses involves the use of digital filters based on the Kalman algorithm [1]. The Kalman filter is a recursive, digital filter which uses models for the measurement and optimal estimates of the system states as well as errors associated with those estimates. In essence, these filters make use of empirical models of analyte responses, as well as any available theory, to enable the resolution of overlapped responses from the chemical components of a mixture. The recursive nature of these filters makes them attractive for “on-line” analysis, because they can process data point-by-point, using only points with high information content, and because concentration estimates can be developed in “real time.” There have already been a number of applications in analytical chemistry [2].

Because these filters are not demanding of memory, and because the simpler forms do not require extensive matrix manipulations, they are easy to implement on a small, low-cost computer. More complex filters can be developed to detect, and even to correct, errors in the chemical models used for the multicomponent analysis. These filters, as well as other “quality assurance” software, can be run as hierarchical filters, with the simple filters acting as intelligent data collectors, and the more complex large filters monitoring the performance of the multicomponent analysis accomplished by the simpler filters. These methods are also easily merged with other chemometric methods, such as factor analysis, to permit more complex, off-line calculations as necessary to provide for improved calibration models, or to model new chemical systems planned for routine study.

## 3. Three Applications of Digital Filters in Multicomponent Analysis

**Figure 1:**
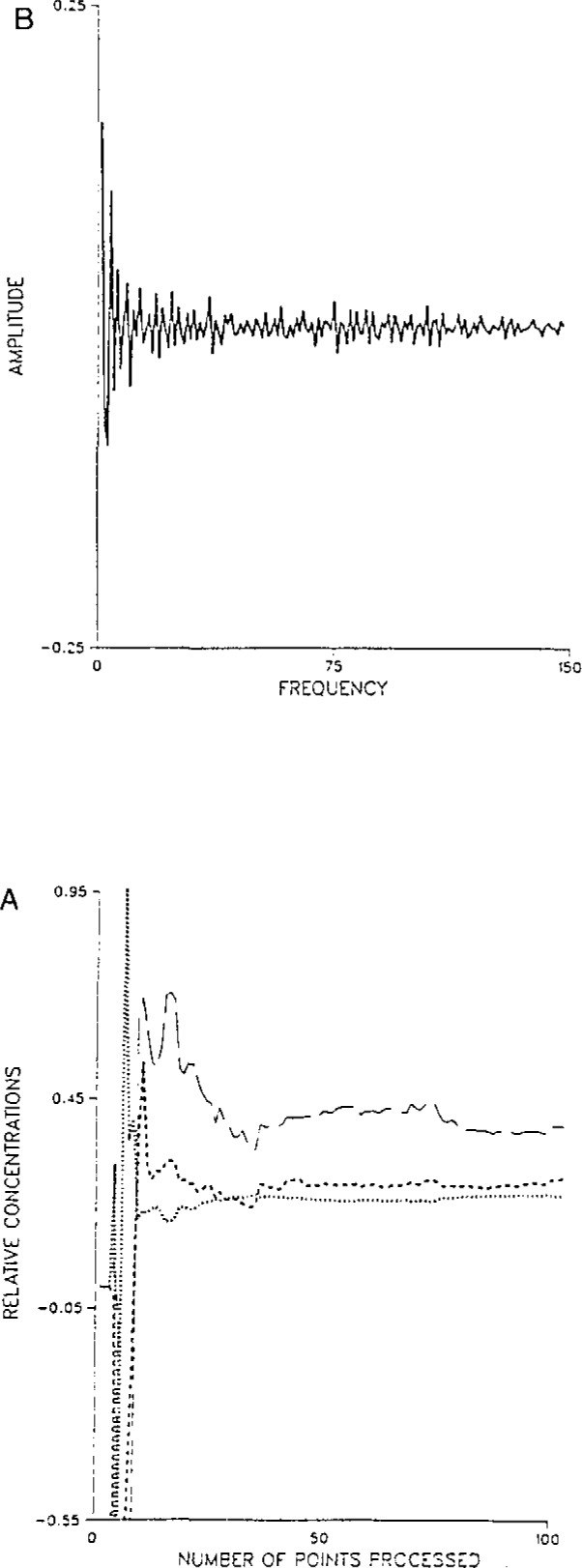
Fourier-domain filtering of spectroscopic data. **A.** Evolution of states for Kalman filtering of multicomponent infrared data. **B**. Portion of interferogram used in filtering multicomponent data.

This paper reports some applications of three new Kalman filters. The first application demonstrates the feasibility of filter-based multicomponent analysis in the Fourier domain, or in fact any other domain reached by a linear transformation from the time domain. Multicomponent analysis in these domains has a number of advantages for real-time analysis: the nature of model errors is changed, and “on-line” FTIR-based multicomponent analysis is made feasible. Figure 1 illustrates filtering of FTIR data in the Fourier domain.

**Figure 2:**
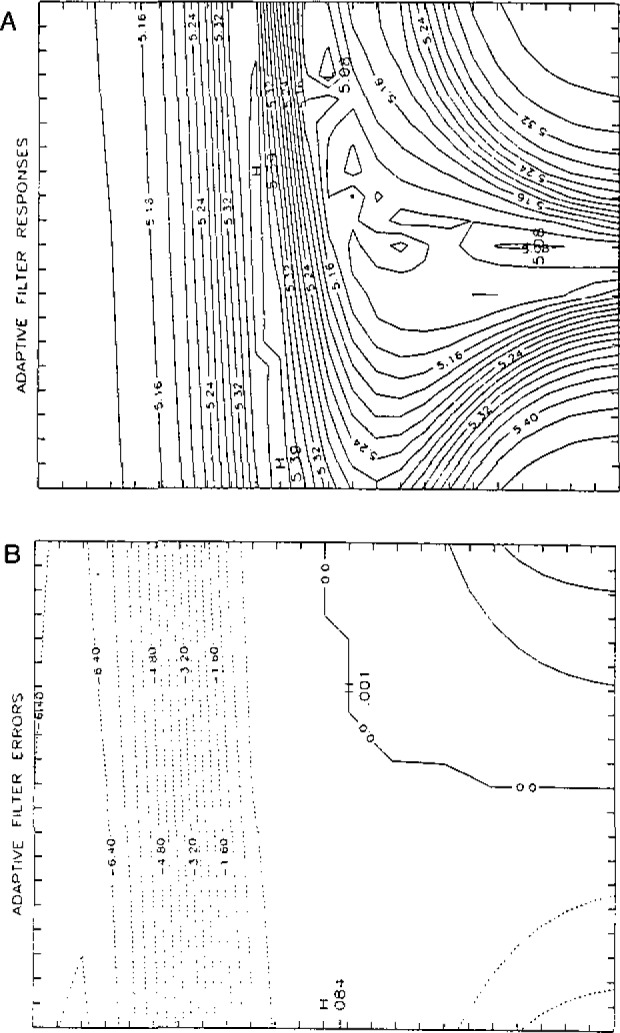
**A.** Entropy surface for innovations from a covariance-matched adaptive filter. The ordinate is the initial guess for the concentration of the modelled component. The abscissa is the initial guess of the covariance matrix element associated with that concentration. **B.** Error surface for estimates produced from a covariance-matched adaptive filter as a function of the initial guesses of the concentration and its covariance. Abscissa and ordinate are as in **A.**

The second application involves the development of new adaptive filters for detection and correction of model error, based on the entropy of the filter innovations. A simplex algorithm is used to drive a sub-optimal adaptive filter to the “best” (minimum entropy) fit, faster and with less error than with other adaptive filters [3]. We have been investigating the convergence properties of this filter. As figure 2 demonstrates, a fairly wide range of initial guesses results in accurate concentration estimates, even when model error occurs *prior* to regions which are correctly modeled in a spectrum.

**Figure 3:**
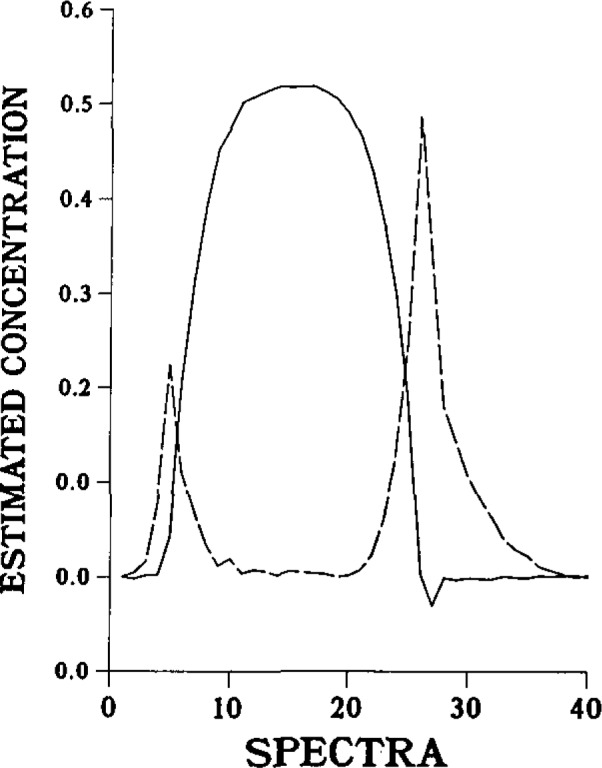
The Kalman filter estimated distribution of nicotinic acid species across an acidic bolus injected into a basic carrier undergoing laminar flow. The dashed line refers to the nicotinate ion, while the solid line refers to nicotinic acid.

The final application involves the “in-line” application of Kalman Filtering for Filtering and quantitation of species observed across the interface created, for example, by injection of a basic solution into a flowing, acidic carrier. These interfaces are typical of those observed in flow injection experiments carried out with low dispersion. Spectroscopic data are collected using diode array spectrometry, and the resulting three-dimensional data are Filtered to obtain concentration estimates for all absorbing species across the interface of carrier and bolus, and across the bolus as well. Figure 3 shows the distribution of species for nicotinic acid and nicotinate ion across an HCl-NaOH interface. The estimated concentrations of the species have less than 2% error without separation or internal standards.

## References

[1] Gelb A (1974). Applied Optimal Estimation.

[2] Brown SD (1986). Anal Chim Acta.

[3] Brown SD, Rutan SC (1985). J Res Natl Bur Stand (US).

